# Supplementary material for: Hsa_circ_0044301 Regulates Gastric Cancer Cell’s Proliferation, Migration, and Invasion by Modulating the Hsa-miR-188-5p/DAXX Axis and MAPK Pathway
Source: Cancers (Basel). 2022 Aug 29;14(17):4183. doi: 10.3390/cancers14174183 (PMC9454757; doi:10.3390/cancers14174183)
Supplement: Supplementary file 1 [file cancers-14-04183-s001.zip › Figure S7.pdf]

Fig S6A left is the original band of HGC-27 actin

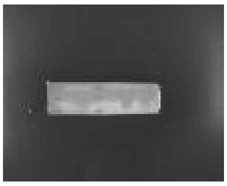

Fig S6A left is the original band of HGC-27 actin-M

FigS6A left is the original band of HGC-27 ERK1/2

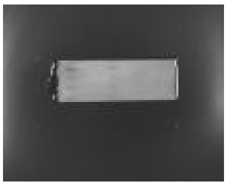

Fig S6A left is the original band of HGC-27 ERK1/2-M

Fig S6A left is the original band of HGC-27 p-ERK

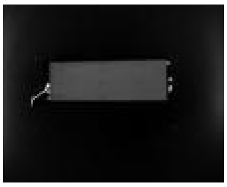

Fig S6A left is the original band of HGC-27 p-ERK-M

Fig S6A original MTOR band of MKN-28 (left) and HGC-27 (right)

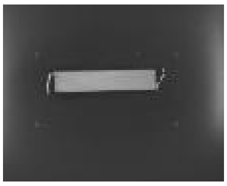

Fig S6A original MTOR-M band of MKN-28 (left) and HGC-27 (right)

Fig S6A left is the original band of MKN-28 actin

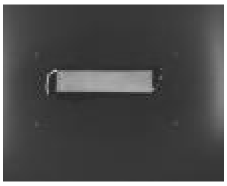

Fig S6A left is the original band of MKN-28 actin-M

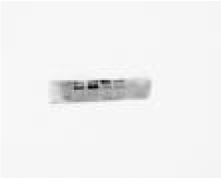

Fig S6A left is the original band of MKN-28 ERK1/2

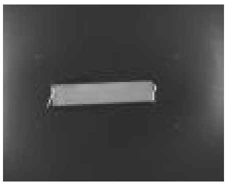

Fig S6A left is the original band of MKN-28 ERK1/2-M

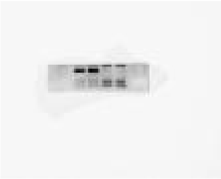

Fig S6A   left is the original band of MKN-28 p-ERK

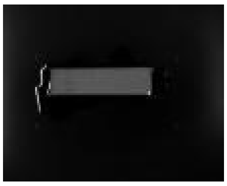

Fig S6A   left is the original band of MKN-28 p-ERK-M

Fig 8B original band of #1 ACTIN

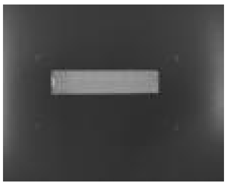

Fig 8B original band of #1 ACTIN-M

Fig 8B original band of #1 ERK1/2

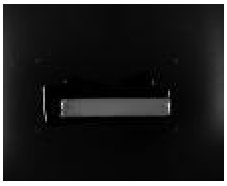

Fig 8B original band of #1 ERK1/2-M

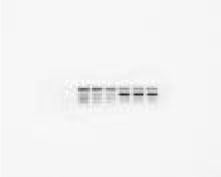

Fig 8B original band of #1 p- ERK (the second line)

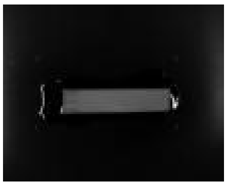

Fig 8B original band of #1 p- ERK-M (the second line)

Fig 8B original band of #2 ACTIN

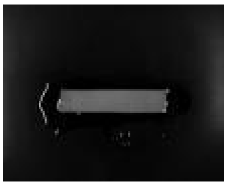

Fig 8B original band of #2 ACTIN-M

Fig 8B original band of #2 ERK1/2

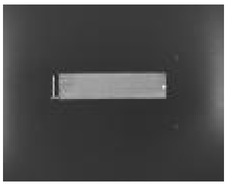

Fig 8B original band of #2 ERK1/2-M

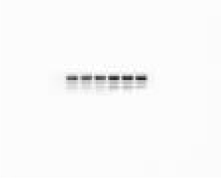

Fig 8B original band of #2 p- ERK

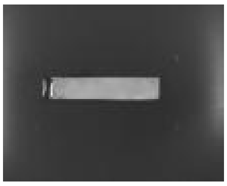

Fig 8B original band of #2 p- ERK-M

Fig 8B original band of #3 ACTIN

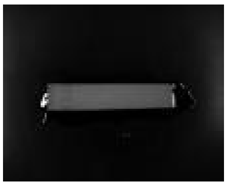

Fig 8B original band of #3 ACTIN-M

Fig 8B original band of #3 ERK1/2

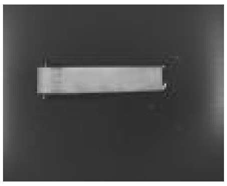

Fig 8B original band of #3 ERK1/2-M

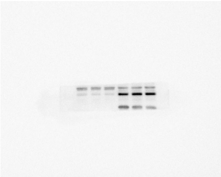

Fig 8B original band of #3 p-ERK1/2

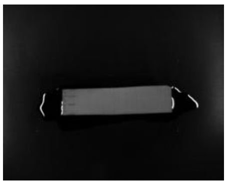

Fig 8B original band of #3 p-ERK1/2-M

Fig 8B original band of #4 ACTIN

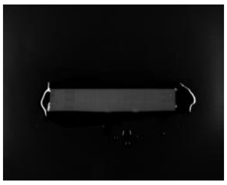

Fig 8B original band of #4 ACTIN-M

Fig 8B original band of #4 ERK1/2

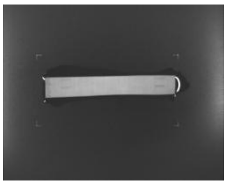

Fig 8B original band of #4 ERK1/2-M

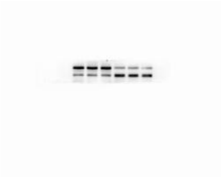

Fig 8B original band of #4 p-ERK1/2

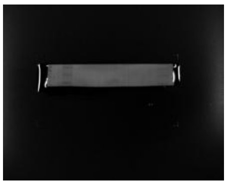

Fig 8B original band of #4 p-ERK1/2-M

Fig 8D original band of ACTIN

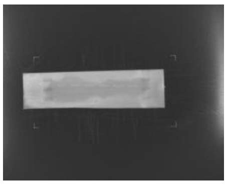

Fig 8D original band of ACTIN-M

Fig 8D original band of ERK1/2

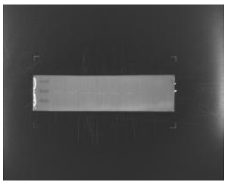

Fig 8D original band of ERK1/2-M

Fig S6C original band of Tubulin

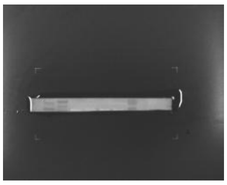

Fig S6C original band of Tubulin-M

Fig S6C original band of ERK1/2

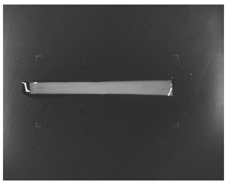

Fig S6C original band of ERK1/2-M

Fig S5C original band of ACTIN

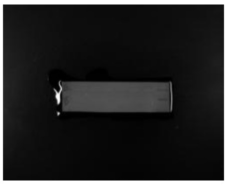

Fig S5C original band of ACTIN-M

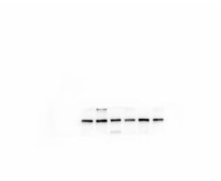

Fig S5C original band of DAXX

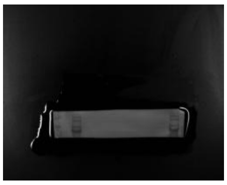

Fig S5C original band of DAXX-M
