# Supplementary figures and images for: Hsa_circ_0044301 Regulates Gastric Cancer Cell’s Proliferation, Migration, and Invasion by Modulating the Hsa-miR-188-5p/DAXX Axis and MAPK Pathway
Source: Cancers (Basel). 2022 Aug 29;14(17):4183. doi: 10.3390/cancers14174183 (PMC9454757; doi:10.3390/cancers14174183)

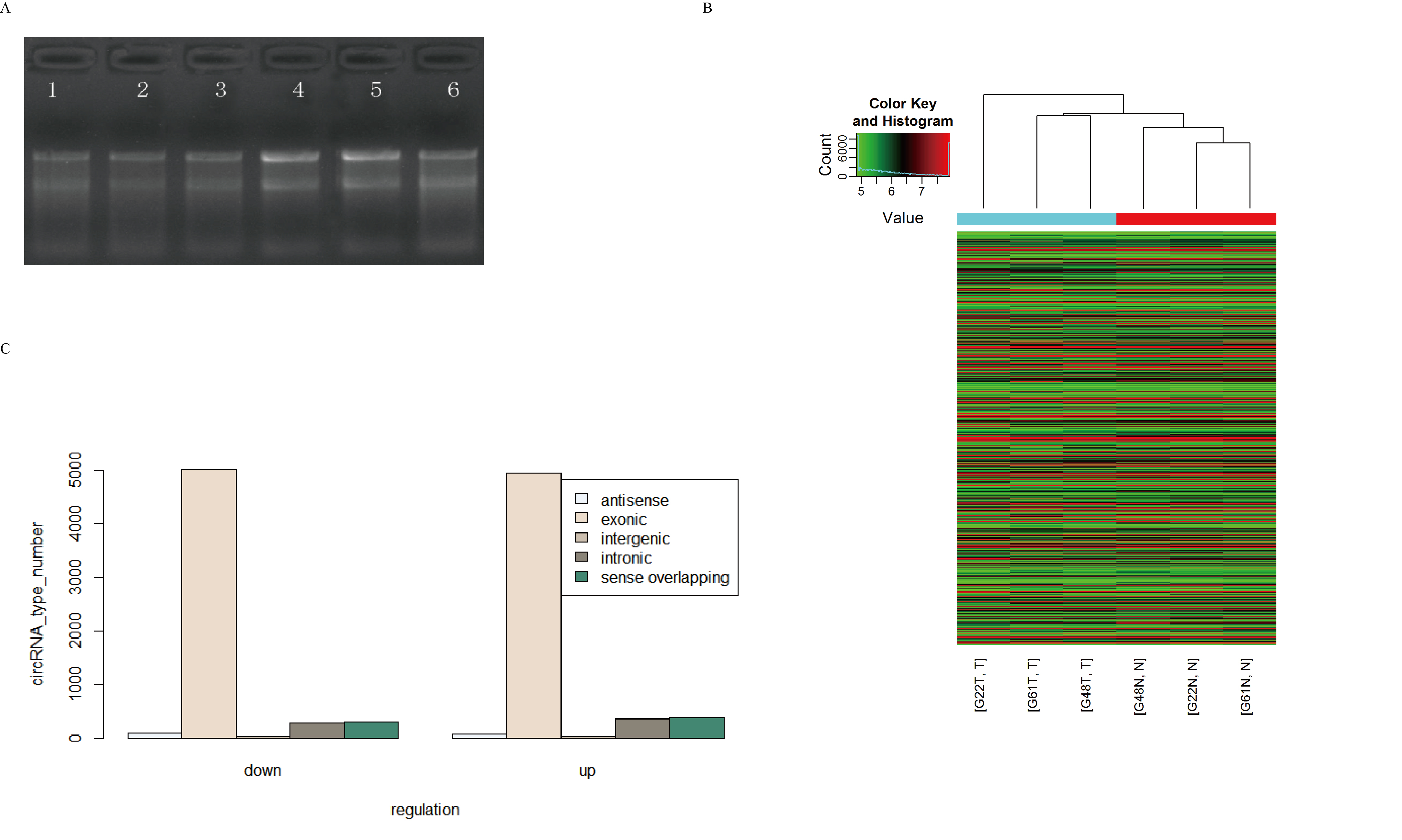

Supplement: Supplementary file 1 [file cancers-14-04183-s001.zip › Figure S1.jpg]

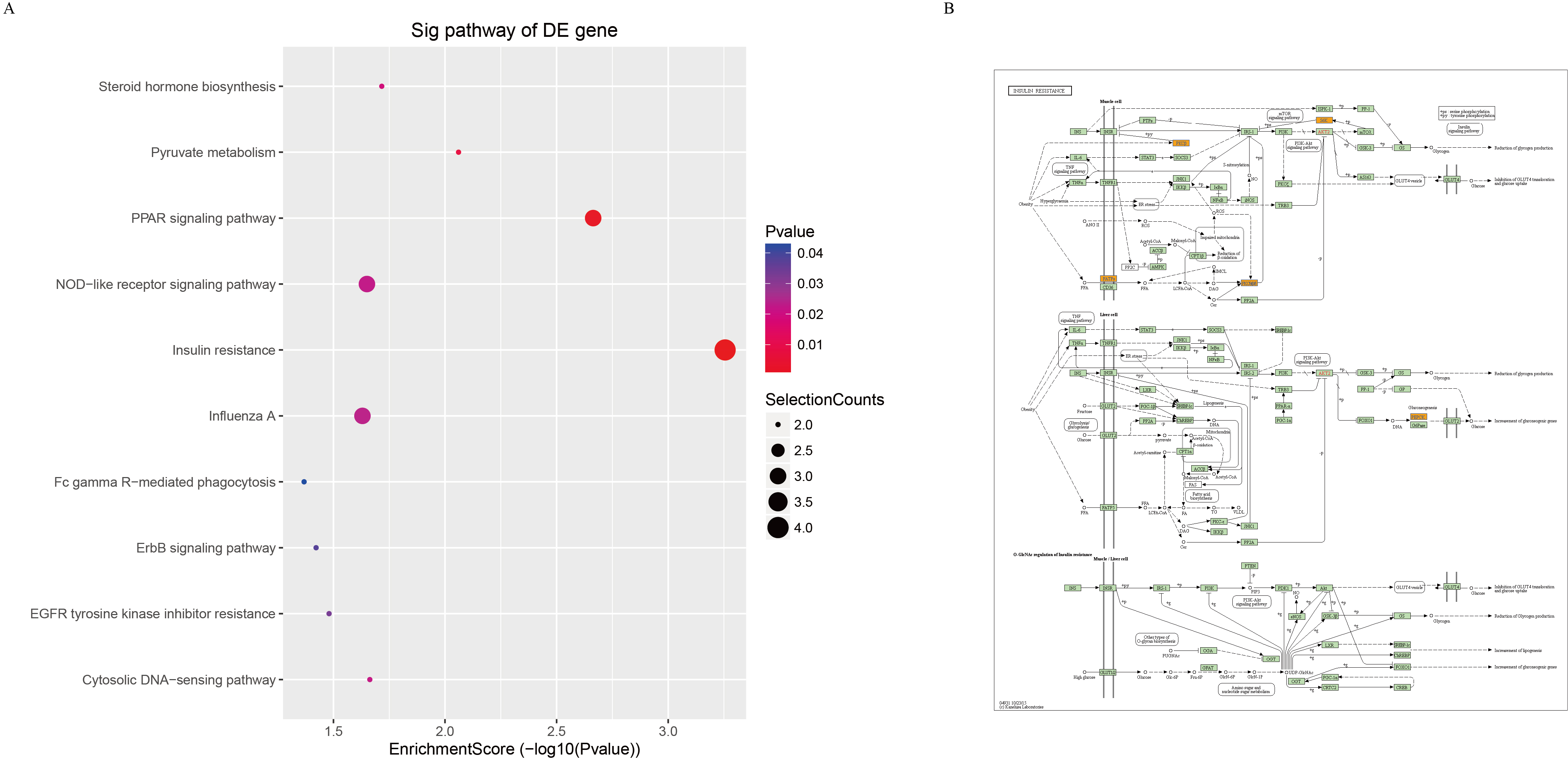

Supplement: Supplementary file 1 [file cancers-14-04183-s001.zip › Figure S2.jpg]

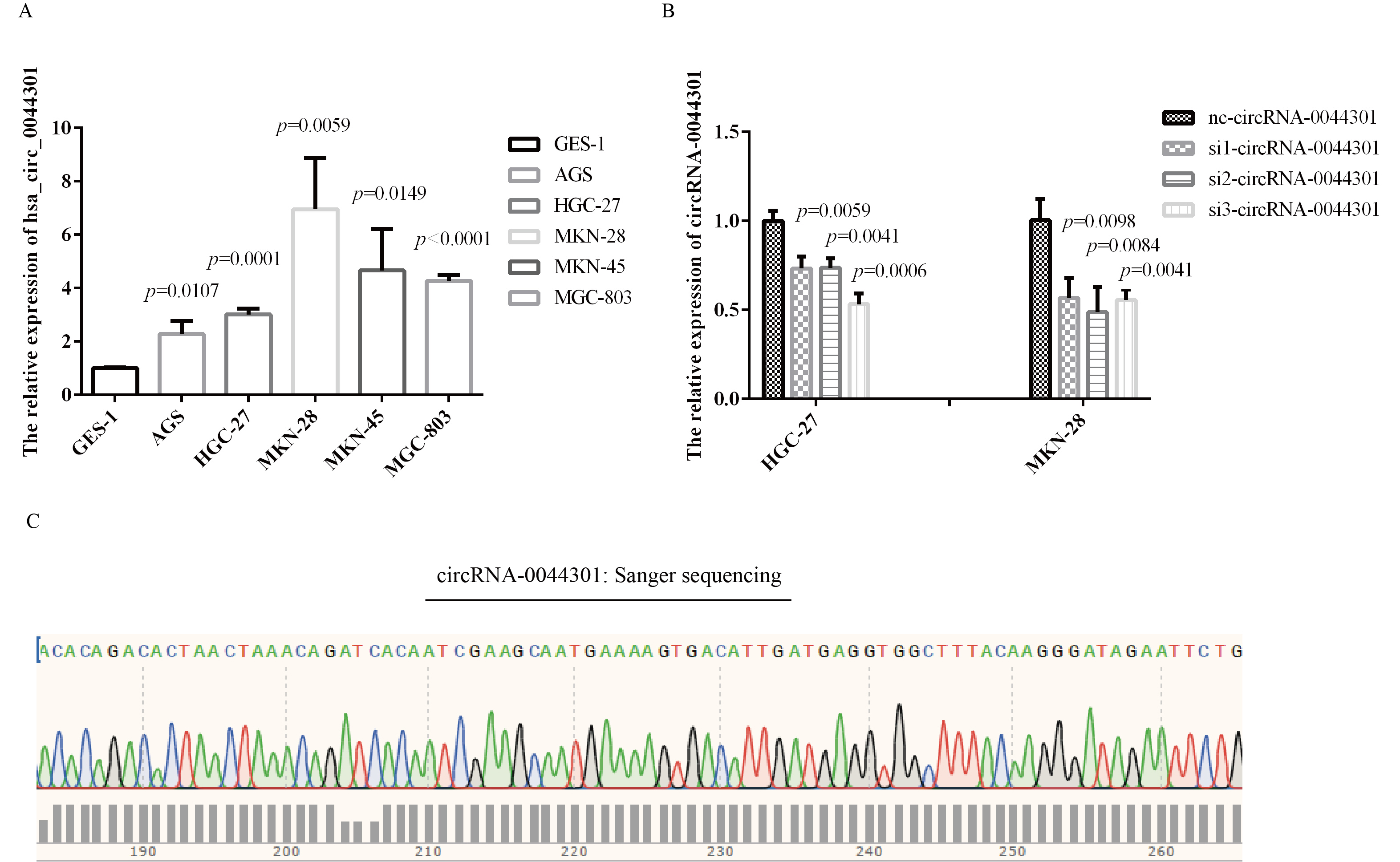

Supplement: Supplementary file 1 [file cancers-14-04183-s001.zip › Figure S3.jpg]

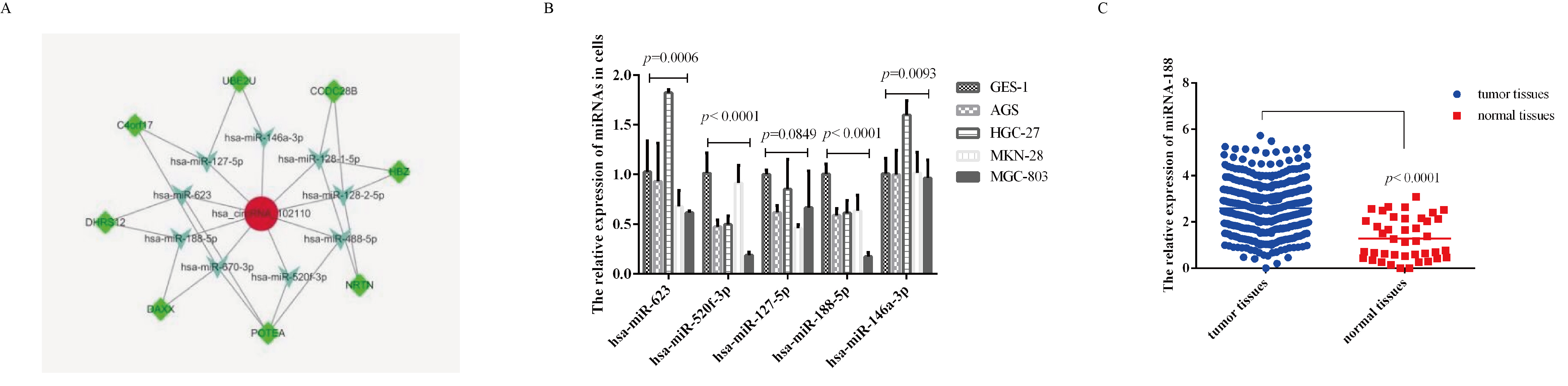

Supplement: Supplementary file 1 [file cancers-14-04183-s001.zip › Figure S4.jpg]

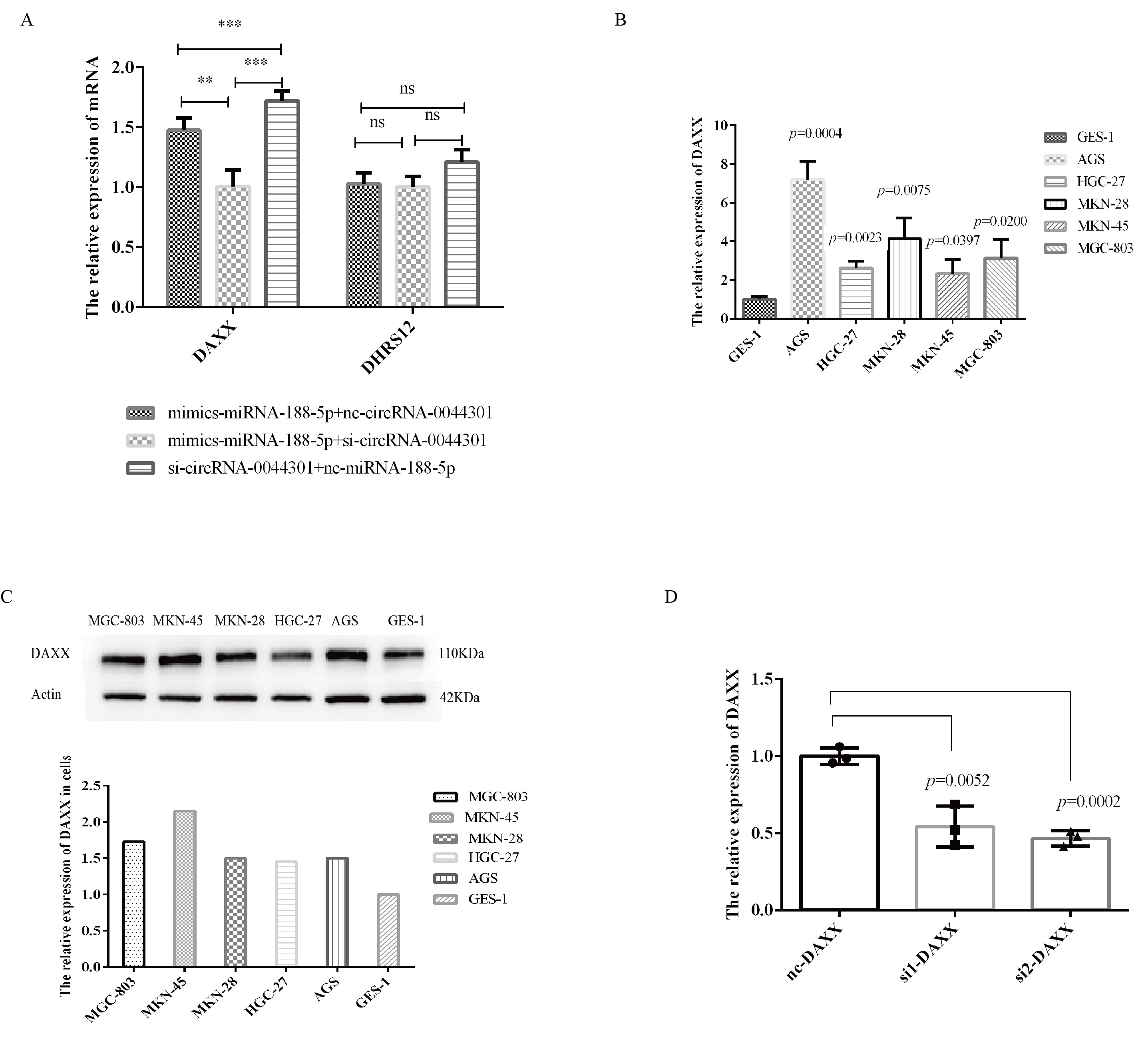

Supplement: Supplementary file 1 [file cancers-14-04183-s001.zip › Figure S5.jpg]

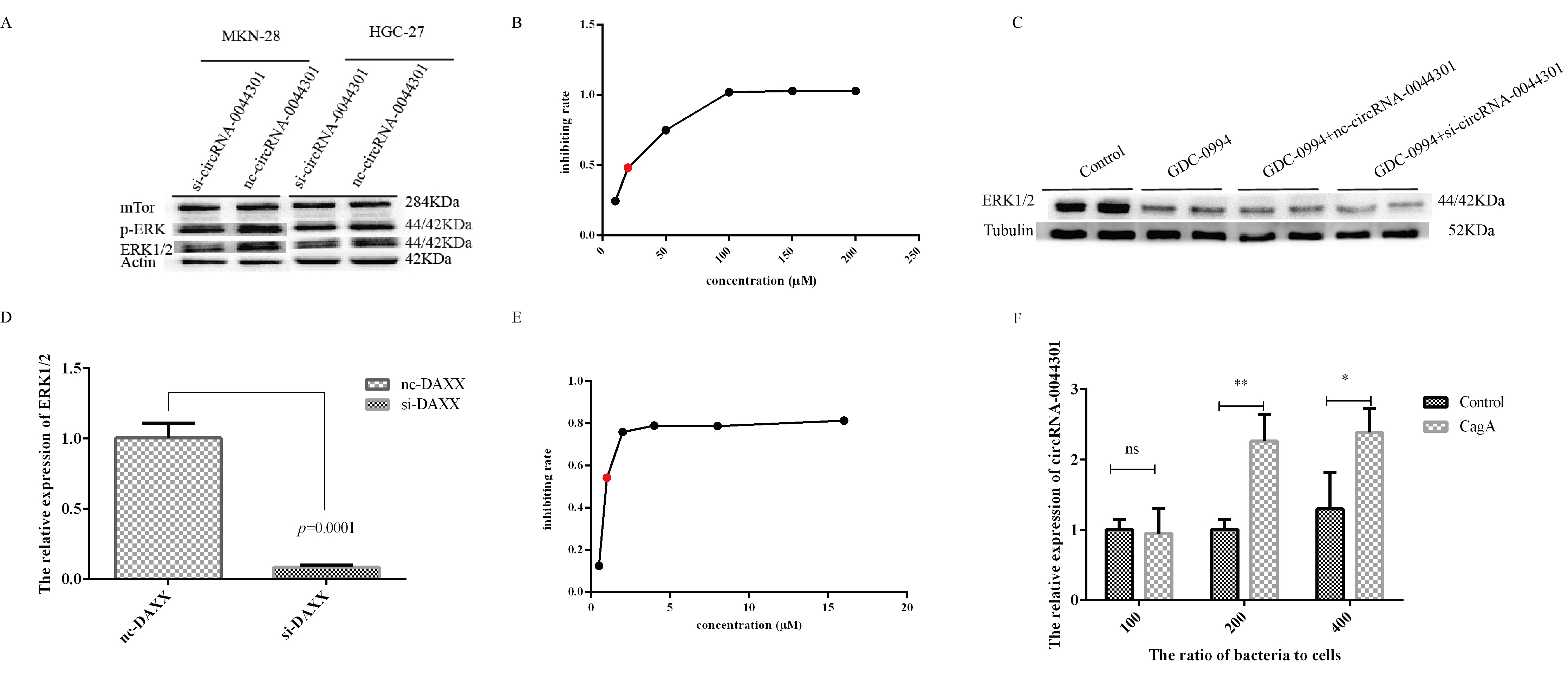

Supplement: Supplementary file 1 [file cancers-14-04183-s001.zip › Figure S6.jpg]
